# Robust dual‐module velocity‐selective arterial spin labeling (dm‐VSASL) with velocity‐selective saturation and inversion

**DOI:** 10.1002/mrm.29513

**Published:** 2022-11-06

**Authors:** Jia Guo

**Affiliations:** ^1^ Department of Bioengineering University of California Riverside Riverside California USA

**Keywords:** arterial spin labeling, diffusion attenuation, eddy current, SNR efficiency, velocity‐selective inversion, velocity‐selective saturation

## Abstract

**Purpose:**

Compared to conventional arterial spin labeling (ASL) methods, velocity‐selective ASL (VSASL) is more sensitive to artifacts from eddy currents, diffusion attenuation, and motion. Background suppression is typically suboptimal in VSASL, especially of CSF. As a result, the temporal SNR and quantification accuracy of VSASL are compromised, hindering its application despite its advantage of being delay‐insensitive.

**Methods:**

A novel dual‐module VSASL (dm‐VSASL) strategy is developed to improve the SNR efficiency and the temporal SNR with a more balanced gradient configuration in the label/control image acquisition. This strategy applies for both VS saturation (VSS) and VS inversion (VSI) labeling. The dm‐VSASL schemes were compared with single‐module labeling and a previously developed multi‐module schemes for the SNR performance, background suppression efficacy, and sensitivity to artifacts in simulation and in vivo experiments, using pulsed ASL as the reference.

**Results:**

Dm‐VSASL enabled more robust labeling and efficient backgroud suppre across brain tissues, especially of CSF, resulting in significantly reduced artifacts and improved temporal SNR. Compared to single‐module labeling, dm‐VSASL significantly improved the temporal SNR in gray (by 90.8% and 94.9% for dm‐VSS and dm‐VSI, respectively; *P* < 0.001) and white (by 41.5% and 55.1% for dm‐VSS and dm‐VSI, respectively; *P* < 0.002) matter. Dm‐VSI also improved the SNR of VSI by 5.4% (*P* = 0.018).

**Conclusion:**

Dm‐VSASL can significantly improve the robustness of VS labeling, reduce artifacts, and allow efficient background suppression. When implemented with VSI, it provides the highest SNR efficiency among VSASL methods. Dm‐VSASL is a powerful ASL method for robust, accurate, and delay‐insensitive perfusion mapping.

## 1 INTRODUCTION

Velocity‐selective arterial spin labeling (VSASL)^1^ is a category of arterial spin labeling (ASL)^2^, ^3^ methods that label arterial blood based on its velocity. Compared to the other 2 ASL categories relying on spatial labeling, and therefore sensitive to arterial transit time (ATT) effects,^4^, ^5^, ^6^, ^7^ that is, pulsed ASL (PASL)^8^, ^9^, ^10^, ^11^ and (pseudo‐) continuous ASL ([P‐]CASL),^3^, ^12^, ^13^ VSASL is insensitive to ATT effects^1^, ^14^ and has an SNR advantage^15^ when arterial blood supply is significantly delayed.

VSASL can be performed with VS saturation (VSS) or VS inversion (VSI) labeling. In VSS,^1^, ^16^, ^17^ ASL signal is created by saturating the magnetization of spins moving above a cutoff velocity (V_cut_) under the label condition and leaving the same population of spins at equilibrium (relaxed) under the control condition. In VSI,^18^, ^19^, ^20^ the blood moving above the V_cut_ is inverted under the control condition and is relaxed under the label condition. The velocity selectivity is realized by the combined effects of RF and flow‐sensitive gradient pulses and a physical mixing process. In both VSS‐ and VSI‐based VSASL, a vascular crushing module (VCM) with the same V_cut_ is required to define the trailing edge of the bolus by removing intravascular signal moving faster than V_cut_ and unwanted venous signal for quantification. A post‐labeling delay (PLD) is the time between the VCM and the image acquisition. In practice, PLD in VSASL is typically set to a minimal value, such as zero, to reduce the T_1_ decay.

Despite recent advancement in VSASL method development,^15^ 2 major challenges remain: (1) the labeling efficiency is relatively low; and (2) artifacts compromise the robustness and the quantification accuracy of VSASL.

For labeling efficiency, the VSS‐based VSASL has a maximal labeling efficiency of 0.5 theoretically, lower than typical values of PASL (0.97) and pseudo‐continuous ASL (PCASL) (0.85) in practice,^21^ resulting in compromised SNR. To improve the SNR, 2 strategies have been developed including: (1) using multiple VSS modules to re‐label relaxed ASL signal and generate a larger labeling bolus^14^; and (2) using a VSI preparation, which has a maximal labeling efficiency of 1 in theory.^20^ In practice, both strategies can improve the SNR by 20%–30% compared to single‐module VSS‐based VSASL.^22^ Despite these achievements, further improvement of the SNR of VSASL is desired.

The artifacts in VSASL mainly come from the fact that the application of flow‐sensitive gradient pulses differs under the label and control conditions. Typically, under the label condition, gradient pulses with zero zeroth moment and non‐zero first moment are applied; whereas under the control condition, zero zeroth and zero first moment are required, either by turning off the gradient pulses or using flow‐compensated gradient pulses. Typically, the gradient pulses under the label and control conditions have small but different diffusion attenuation^1^, ^14^ and different sensitivity to eddy current (EC) effects.^16^, ^17^ Such difference makes the labeling sensitive to processes that are irrelevant to blood flow, such as diffusion attenuation, or undesired labeling of tissues caused by ECs; that is, artifactual ASL “signals” are generated, resulting in compromised robustness and quantification accuracy. For example, cerebral blood flow (CBF) may be significantly overestimated if EC effects are not reduced or properly matched in the label and control images.^16^, ^17^ Methods have been developed to improve the preparation and quantification accuracy of VSASL, such as reducing sensitivity to EC effects,^16^, ^17^ and correction of artifactual ASL signal due to diffusion attenuation effects, especially in voxels containing CSF.^14^ Despite these development and research efforts, the temporal SNR (tSNR) of existing VSASL methods is still low in practice.^20^, ^22^, ^23^

In this study, a novel dual‐module labeling strategy is developed to address the 2 major challenges in VSASL described above. It is applicable with VSS and VSI labeling modules and their combinations. In addition, it also enables better background suppression (BS)^24^ than existing VSASL methods, further enhancing the SNR performance. To differentiate the new dual‐module labeling method from the previous VSS‐based multi‐module VSASL (mm‐VSASL) method,^14^ we refer to the new dual‐module labeling strategy as dual‐module VSASL (dm‐VSASL), though the mm‐VSASL can be (and typically is) implemented with 2 VSS modules.

The principles of the dm‐VSASL scheme are first introduced and followed by the modeling of dm‐VSASL signal and the optimization for maximal SNR efficiency. The practical performance, including the ASL signal strength, labeling robustness or tSNR, BS performance, and CBF quantification, was examined and compared with existing VSASL methods and PASL in in vivo experiments.

## 2 THEORY

As described above, traditional VSASL (single‐module VSASL, or sm‐VSASL) has different gradient layouts, and thus unbalanced diffusion and EC sensitivities, in the acquisition of label and control images; and mm‐VSASL has improved SNR efficiency, but the diffusion and EC sensitivities are higher; that is, 2 VS modules under the label condition (with flow‐sensitizing gradients) are used to acquire label images. To tackle this, the dm‐VSASL design rearranges the flow‐sensitizing gradients in the acquisition of label and control images such that the diffusion and EC sensitivities are better balanced in the 2; therefore, the associated artifacts can be reduced or canceled after subtraction. Similar to mm‐VSASL, dm‐VSASL uses more than 1 VS labeling module in preparation, but they differ in a few important aspects: (1) mm‐VSASL is applicable with VSS labeling only, whereas dm‐VSASL can use both VSS and VSI labeling and their combinations; (2) dm‐VSASL uses a different gradient configuration to acquire label and control images; (3) dm‐VSASL requires the first VS module to invert the static spins, whereas mm‐VSASL does not.

**FIGURE 1:**
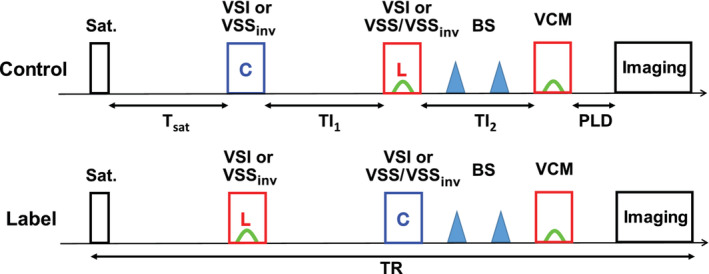
The new dual‐module VSASL scheme, where the VS modules under the control condition are labeled with C (blue), and the ones under the label condition are labeled with L (red), with green shapes representing flow‐sensitive gradient pulses. The pulse sequence diagram on the top is for acquiring a control image and the diagram at the bottom for acquiring a label image. TI_1_ and TI_2_ are the time between the 2 VS modules, and between the second VS module and the VCM. BS, background suppression; PLD, post‐labeling delay; VCM, vascular crushing module; VSASL, velocity‐selective arterial spin labeling; VSI, velocity‐selective inversion; VSS, velocity‐selective saturation; VSS_inv_, velocity‐selective saturation that can invert stationary magnetization.

Below we start with the implementation of dm‐VSASL to demonstrate the design principles as illustrated in Figure 1. Briefly, a global saturation is first applied to reset the magnetization of all spins to a known state ($M_{z}=0$). After a delay time of $T_{\mathrm{sat}}$ (e.g., 2 s) for the arterial spins to recover, 2 VS modules are applied consecutively with a delay (on the order of 1 s) after each to allow arterial inflow to deliver. The types of the VS modules (VSS vs. VSI) and the conditions (label vs. control) depend on the specific implementation and the image type being acquired (label vs. control), as described in the details below. Then a VCM is applied to remove undelivered (i.e., with V > V_cut_) spins and followed by image acquisition after a short PLD (close to zero). Additional BS pulses can be applied between the second VS module and the VCM.

### 2.1 Dm‐VSASL using VSI only

To acquire a control image, the first VSI module is applied under the control condition, that is, without flow‐sensitive gradients. After a delay of TI_1_, the second VSI module is applied under the label condition, that is, with the flow‐sensitive gradient pulses. After a second delay time TI_2_, the VCM can be applied and followed by PLD and image acquisition. To acquire a label image, the first VSI module is applied under the label condition, and the second module under the control condition. Background tissue signals are partially suppressed by the inversion effect of the VSI modules, and the SNR is improved.^22^ Additional global BS pulses can be applied after the second VSI module to further improve the SNR.

### 2.2 Dm‐VSASL using VSS only

Unlike the previous mm‐VSASL, where 2 VSS modules under the same condition are applied consecutively to acquire a label or a control image, dm‐VSASL using VSS only obtains a control image with the first VSS module under the control condition and the second module under the label condition, and a label image with the first VSS module under the label condition and the second module under the control condition. In addition, the first VSS module has to be modified to invert the magnetization of static spins. To differentiate it from the unmodified VSS module, we refer it to as VSS_inv_. The second VSS module can be either VSS or VSS_inv_.

VSS_inv_ can be implemented in 2 ways: (1) applying an inversion pulse immediately after the VSS module, or (2) modifying the phase of the RF pulses in the VSS module to induce a built‐in inversion effect as described in Ref. 25; for example, a phase of π can be added to the last RF pulse in a double‐refocused hyperbolic secant/tangent^1^ or a symmetric 8‐segment B_1_ insensitive rotation (sBIR8)^16^ module to tip the static spins down instead of up. The VSS_inv_ module with built‐in inversion is preferred because: (1) it does not increase the specific absorption rate; and (2) no addition signal reduction is introduced. Like VSI, VSS_inv_ effectively serves as a BS pulse whose inversion effect should be accounted for in BS timing calculation.

### 2.3 Dm‐VSASL using both VSS and VSI

Combinations of VSS and VSI modules, such as VSI + VSS/VSS_inv_ and VSS_inv_ + VSI, are also feasible. For example, a VSI module followed by a VSS or VSS_inv_ module, or a VSS_inv_ followed by a VSI module, would also work under the principles of dm‐VSASL.

Note that for the dm‐VSASL implementations described above, the label/control condition switching is required for proper accumulation of ASL signal (see below). Otherwise, the ASL signal created by the 2 VS modules will have opposite signs, resulting in signal reduction or even cancellation, as in the examples shown in Supporting Information Figure S1.

Compared to sm‐VSASL and mm‐VSASL, dm‐VSASL has a more balanced gradient configuration between the label and the control image acquisition. This arrangement should mitigate the eddy current and the diffusion attenuation effects that are typically observed in VSASL, as well as reducing its sensitivity to motion, potentially reducing artifacts and improving quantification accuracy. In addition, the inversion effects at an early time allow more flexible and efficient BS. All these should contribute to improving the labeling stability, the tSNR, and the quantification accuracy of VSASL.

### 2.4 Dm‐VSASL signal modeling and blood flow quantification

**FIGURE 2:**
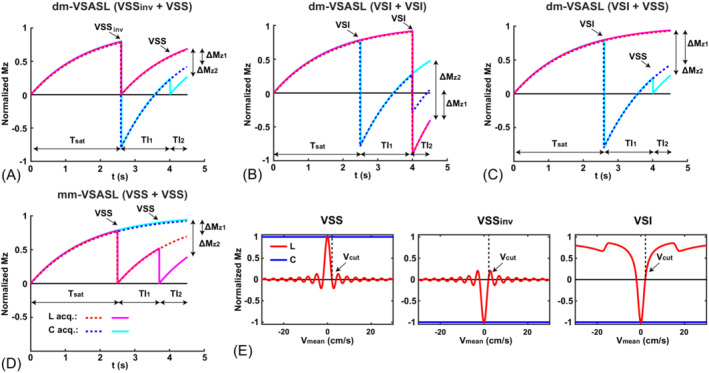
The magnetization evolution of the *proposed* dm‐VSASL with label/control switching (Figure 1) using VSS_inv_ + VSS (A), VSI + VSI (B) and VSI + VSS (C); and the *pervious* mm‐VSASL (no label/control switching) using VSS + VSS (D); (E) the M_z_ vs. V_mean_ profiles of VSS, VSS_inv_ and VSI labeling after convolving with a laminar flow distribution with the V_cut_ shown(label: red, control: blue). In panels A–D, the group of arterial spins seeing only the first VS module is shown in dashed lines (group 1), and the group seeing both VS modules is shown in solid lines (group 2). The magnetization evolution in the label image acquisition is shown in warm colors (group 1: dashed red; group 2: solid magenta), and that in the control image acquisition is shown in cool colors (group 1: dashed blue; group 2: solid cyan). The curves in A–D are shifted slightly for better visualization, and only T_1_ relaxation effect was included. Note that the label/control condition in the second VS module is switched in A, B and C, such that the ASL signals from groups 1 and 2 (ΔM_z1_and ΔM_z2_) are of the same sign.

Similar to the previous mm‐VSASL signal modeling,^14^ 3 groups of arterial spins are considered in dm‐VSASL: (1) group 1 being labeled by only the first VS module, that is, it is in the transmit field of the RF coil and moves above V_cut_ at the application of the first VS module and has decelerated below V_cut_ (delivered) at the application of the second VS module; (2) group 2 being labeled by both VS modules, that is, in the range of the RF coil and moving above V_cut_ at the application of both VS modules; and (3) group 3 being labeled only by the second VS module, that is, moving into the transmit field of the RF coil after the first VS module. Since group 3 is not likely to contribute to the measured ASL signal when ${\mathrm{TI}}_{1}+{\mathrm{TI}}_{2}<{\mathrm{BD}}_{\max}$ (${\mathrm{BD}}_{\max}$ is the maximal bolus duration, on the order of 2 s) and including it complicates the quantification,^14^ only the first 2 groups are included in the following modeling. The evolution of the magnetization of the 2 groups is shown in Figure 2. Note that the label/control condition switching in the second VS module is necessary to ensure the ASL signals from the 2 groups are of the same sign.

With ideal VS modules, that is, without considering the labeling efficiency, the ASL signal can then be modeled as

(1) $$\begin{array}{cc} {\mathrm{Sig}}_{\mathrm{ASL}} & ={\mathrm{Sig}}_{\text{ctrl}}-{\mathrm{Sig}}_{\mathrm{lab}}\\ & =f\cdot \left(∆M_{z1}\cdot {\mathrm{TI}}_{1}+∆M_{z2}\cdot {\mathrm{TI}}_{2}\right)\cdot e^{-\frac{\mathrm{PLD}}{T_{1a}}}, \end{array}$$

where f is the blood flow under investigation; $∆M_{z1}$ and $∆M_{z2}$ are the magnetization difference of arterial spin groups 1 and 2 between the control and label acquisition (${\mathrm{Sig}}_{\text{ctrl}}-{\mathrm{Sig}}_{\mathrm{lab}}$) without considering labeling efficiency; ${\mathrm{TI}}_{1}$ and ${\mathrm{TI}}_{2}$ are the time between the first and the second VS modules, and that between the second VS and the VCM modules; and $T_{1a}$ is the $T_{1}$ of arterial blood.

When realistic VS pulses are considered, the 2 groups of arterial spins are affected differently by the labeling efficiency and the T_2_ relaxation of the VS modules. The labeling efficiency of a VS module $\alpha_{\mathrm{VS}}$ can be modeled as^22^:

(2) $$\alpha_{\mathrm{VS}}=\beta \cdot e^{-\frac{{\mathrm{eTE}}_{\mathrm{VS}}}{T_{2a}}},$$

where ${\mathrm{eTE}}_{\mathrm{VS}}$ is the effective TE of the VS module; $T_{2a}$ is the arterial T_2_ relaxation time; and β is a scaler containing other factors affecting the VS labeling efficiency, such as the shape of the VS labeling profile with respect to velocity, distribution of arterial blood velocity, and sensitivity to B_0_ and B_1_ variations within the labeling volume. Note that for VSS, a factor of 0.5 is not included, as the $\alpha_{\mathrm{VS}}$ is defined based on the $∆M_{z}$ created by VS preparation (see below), not normalized by $2M_{0}$ as defined in Reference 15.

Under such definition, group 1 is first affected by the labeling efficiency of the first VS module $\alpha_{\mathrm{VS}1}$, and then only scaled by the T_2_ relaxation of the second VS module since the arterial blood has decelerated and reached the capillary bed when the second VS module is applied; whereas group 2 is affected by both VS modules as it is in the arterial space at the application of both VS modules. Following Equation 1, the magnetization difference of the 2 groups can be approximated (see Supporting Information for detailed derivation) as:

For dm‐VSASL using VSI + VSI:

$$\begin{array}{cc} & ∆M_{z1}=2M_{0}\cdot e^{-\frac{{\mathrm{eTE}}_{\mathrm{VSI}}}{T_{2a}}}\cdot \alpha_{\mathrm{VSI}}\cdot e^{-\frac{{\mathrm{TI}}_{1}+{\mathrm{TI}}_{2}}{T_{1a}}}\cdot \left(1-e^{-\frac{T_{\mathrm{sat}}}{T_{1a}}}\right),\;\text{and}\\ & ∆M_{z2}=2M_{0}\cdot \alpha_{\mathrm{VSI}}\cdot e^{-\frac{{\mathrm{TI}}_{2}}{T_{1a}}}\cdot \left(1-e^{-\frac{{\mathrm{TI}}_{1}}{T_{1a}}}\right), \end{array}$$

where $T_{\mathrm{sat}}$ is the time between the global saturation and the first VS module, and $M_{0}$ is the fully relaxed magnetization of arterial blood. Note the absence of the $\alpha_{\mathrm{VSI}}^{2}$ terms in $∆M_{z2}$ due to complete cancelation from the inversion in VSI and the label/control switching.

For dm‐VSASL using VSS_inv_ + VSS:

$$\begin{array}{cc} & ∆M_{z1}=-M_{0}\cdot e^{-\frac{{\mathrm{eTE}}_{\mathrm{VSS}}}{T_{2a}}}\cdot \alpha_{\mathrm{VSS}}\cdot e^{-\frac{{\mathrm{TI}}_{1}+{\mathrm{TI}}_{2}}{T_{1a}}}\cdot \left(1-e^{-\frac{T_{\mathrm{sat}}}{T_{1a}}}\right),\;\text{and}\\ & ∆M_{z2}=-M_{0}\cdot \alpha_{\mathrm{VSS}}\cdot e^{-\frac{{\mathrm{TI}}_{2}}{T_{1a}}}\cdot \left(1-e^{-\frac{{\mathrm{TI}}_{1}}{T_{1a}}}\right). \end{array}$$

Note that there is no $\alpha_{\mathrm{VSS}}^{2}$ term accumulated in $∆M_{z2}$ because the label condition is applied in both the label and control image acquisition, which sets $M_{z}$ to 0, that is, removing the effect of $\alpha_{\mathrm{VSS}}$.

For reference, the mm‐VSASL using VSS + VSS has

$$\begin{array}{cc} & ∆M_{z1}=M_{0}\cdot e^{-\frac{{\mathrm{eTE}}_{\mathrm{VSS}}}{T_{2a}}}\cdot \alpha_{\mathrm{VSS}}\cdot e^{-\frac{{\mathrm{TI}}_{1}+{\mathrm{TI}}_{2}}{T_{1a}}}\cdot \left(1-e^{-\frac{T_{\mathrm{sat}}}{T_{1a}}}\right),\;\text{and}\\ & ∆M_{z2}=M_{0}\cdot \alpha_{\mathrm{VSS}}\cdot e^{-\frac{{\mathrm{TI}}_{2}}{T_{1a}}}\\ & \;\cdot \left(1-e^{-\frac{{\mathrm{TI}}_{1}}{T_{1a}}}+\alpha_{\mathrm{VSS}}\cdot e^{-\frac{{\mathrm{TI}}_{1}}{T_{1a}}}-\alpha_{\mathrm{VSS}}\cdot e^{-\frac{T_{\mathrm{sat}}+{\mathrm{TI}}_{1}}{T_{1a}}}\right). \end{array}$$

And for hybrid dm‐VSASL using VSI + VSS,

$$\begin{array}{cc} & ∆M_{z1}=-2M_{0}\cdot e^{-\frac{{\mathrm{eTE}}_{\mathrm{VSS}}}{T_{2a}}}\cdot \alpha_{\mathrm{VSI}}\cdot e^{-\frac{{\mathrm{TI}}_{1}+{\mathrm{TI}}_{2}}{T_{1a}}}\cdot \left(1-e^{-\frac{T_{\mathrm{sat}}}{T_{1a}}}\right),\;\text{and}\\ & ∆M_{z2}=-M_{0}\cdot \alpha_{\mathrm{VSS}}\cdot e^{-\frac{{\mathrm{TI}}_{2}}{T_{1a}}}\\ & \;\cdot \left(1-e^{-\frac{{\mathrm{TI}}_{1}}{T_{1a}}}+\alpha_{\mathrm{VSI}}\cdot e^{-\frac{{\mathrm{TI}}_{1}}{T_{1a}}}-\alpha_{\mathrm{VSI}}\cdot e^{-\frac{T_{\mathrm{sat}}+{\mathrm{TI}}_{1}}{T_{1a}}}\right). \end{array}$$

For mm‐VSASL and hybrid dm‐VSASL, there are α^2^ terms left due to incomplete cancelation or lack of label/control switching.

Note the sign difference between different dm‐VSASL implementations. Since ${\mathrm{Sig}}_{\mathrm{ASL}}$ is affected by the T_2_ relaxation of both VS modules, it is therefore beneficial to use VS modules with short eTE. For example, sinc‐shaped VSI (sinc‐VSI)^22^ is preferred over rectangular‐shaped VSI (rect‐VSI)^20^ in dm‐VSI for its shorter eTE (29.4 ms versus 37.6 ms with the same pulse duration of ˜64 ms).

## 3 METHODS

### 3.1 Optimizing SNR efficiency of VSASL

One of the major goals of designing dm‐VSASL is to improve its SNR efficiency (SNR per unit time), defined as ${\mathrm{Sig}}_{\mathrm{ASL}}/\sqrt{2\mathrm{TR}}$. A kinetic ASL signal model^26^ was used to model and compare the SNR efficiency of different VSASL schemes, along with PASL and PCASL for reference.^14^ The SNR efficiency was evaluated with ideal or realistic labeling efficiencies, at different TR ranging from 2 to 8 s. At each TR, the maximal SNR efficiency was calculated with different values of TI (for sm‐VSASL), or ${\mathrm{TI}}_{1}$ and ${\mathrm{TI}}_{2}$ (for mm‐VSASL and dm‐VSASL) by grid searching under the constraint $\mathrm{TI}<{\mathrm{BD}}_{\max}$ or ${\mathrm{TI}}_{1}+{\mathrm{TI}}_{2}<{\mathrm{BD}}_{\max}$, respectively. ${\mathrm{BD}}_{\max}$ is the maximal bolus duration: 2 s for VSASL,^14^ 1 s for PASL, and unlimited for PCASL. Other parameters included: $T_{1a}$ = 1.66 s, $T_{\mathrm{Acq}}$ = 0.5 s, ${\mathrm{PLD}}_{\text{PASL}}$ = 1.6 s, ${\mathrm{PLD}}_{\text{PCASL},1}$ = 1.8 s, ${\mathrm{PLD}}_{\text{PCASL},2}$ = 2.2 s, $\alpha_{\text{PASL}}=0.98$, and $\alpha_{\text{PCASL}}=0.85.$ ^21^ When T_2_ relaxation was considered for VSASL: $T_{2a}$ = 150 ms, ${\mathrm{eTE}}_{\mathrm{VSS}}$ = 20 ms, ${\mathrm{eTE}}_{\mathrm{VSI}}$ = 30 ms; when realistic *β* was considered: $\beta_{\mathrm{VSS}}$ = 0.9 and $\beta_{\mathrm{VSI}}$ = 0.7 were assumed.^22^

### 3.2 In vivo experiments

Six young healthy subjects (2 female, age 21–38 years) were studied on a 3 Tesla scanner (Siemens Prisma, Erlangen, Germany) under University of California Riverside's Internal Review Board approval and written consent from the subjects. Among the dm‐VSASL implementations, 2 dm‐VSASL with VSS labeling only and VSI labeling only were implemented and tested. sBIR8^16^ and sinc‐VSI^22^ pulses were used for VSS and VSI, respectively. There are other VS pulses, such as BIR‐4^27^ and double‐refocused hyperbolic secant/tangent^1^ for VSS and rect‐VSI^20^ for VSI labeling. sBIR8 VSS was chosen for its B_0_/B_1_ insensitivity and robustness against EC effects,^16^ and sinc‐VSI was chosen for its higher labeling efficiency (shorter effect TE) and smoother velocity‐labeling profile compared to rect‐VSI.^22^

Single‐module VSASL using VSS and sinc‐VSI labeling, and mm‐VSASL using 2 VSS modules, were compared. A PASL scan was also included as the reference for its robust labeling efficiency compared to PCASL in the presence of off‐resonance and blood velocity difference.^12^, ^28^ The following ASL scans with BS were performed in a randomized order in each subject: (1) PASL: FAIR^9^, ^29^ with Q2TIPS,^30^ TI_1_ = 0.8 s, TI = 2.4 s (PLD = 1.6 s), 2 BS pulses applied at 1.4 s and 0.42 s before imaging; (2) sm‐VSASL using VSS (VSS_inv_ or VSS for simplicity): TI = 1.4 s, 1 BS pulse at 0.48 s before imaging; (3) mm‐VSASL (VSS + VSS_inv_ or mm‐VSS): TI_1/2_ = 1.15/0.82 s, 1 BS pulse 0.26 s before imaging; (4) dm‐VSASL using VSS (VSS_inv_ + VSS or dm‐VSS, BS1): TI_1/2_ = 1.45/0.54 s, 1 BS pulse at 0.28 s before imaging; (5) sm‐VSASL using VSI (VSI): TI = 1.4 s, 1 BS pulse at 0.48 s before imaging; (6) dm‐VSASL using VSI (dm‐VSI, BS1): TI_1/2_ = 1.45/0.54 s, 2 BS pulses at 0.47 s and 0.14 s before imaging. To explore the flexibility and the effectiveness of BS in dm‐VSASL, additional scans with different BS timings were performed: (7) dm‐VSASL using VSS (VSS_inv_ + VSS_inv_ or dm‐VSS, BS2), and (8) dm‐VSASL using VSI (dm‐VSI, BS2), both with the same timings: TI_1/2_ = 1.45/0.54 s, 2 BS pulses at 0.37 s and 0.25 s before imaging.

Other imaging parameters included: 2‐segmented (along the slice‐encoding direction) 3D gradient and spin echo (GRASE) EPI readout with 180° refocusing RF pulses; an in‐plane FOV of 220 × 220 mm and a matrix size of 64 × 64; 24 slices and 4 mm thickness to cover the whole brain; TR = 4 s (PASL) and 5 s (VSASL); TE = 36.1 ms; 15 and 12 label/control pairs for PASL and VSASL, respectively. In VSASL, a VCM using sBIR8 VSS module was applied about 100 ms (PLD) before image acquisition. The V_cut_ was 2 cm/s along the superoinferior direction. The total scan time was 4 min for each ASL scan. Additional fully relaxed proton‐density‐weighted reference images were acquired for quantification. 3D T_1w_ anatomical images were collected using MP‐RAGE sequence with TR/TE = 2.4 s/2.72 ms, TI = 1.06 s, an isotropic resolution of 0.8 mm, and an acquisition time of 6.5 min.

### 3.3 Data processing

To ensure the quality of ASL images, the first pair of ASL acquisition was discarded. The BS level (tissue signal) maps were calculated by dividing the mean of control/label images by the relaxed reference images, and expressed in percentage. ASL signal was produced with pairwise subtraction. The signal reductions due to additional BS pulses were corrected, assuming 5% reduction per BS pulse. Normalized mean ASL signal was calculated as percentage relative to the reference image for comparison across subjects. The average ASL signal across time was divided by its temporal SD to calculate the tSNR^31^ in each scan. CBF was quantified with the modeling and parameters provided earlier. Gray and white matter (GM and WM) and CSF regions of interest (ROIs) were identified after registering the T_1w_ anatomical images to the ASL images and segmentation using the FSL toolbox.^32^ The normalized ASL signal, tSNR, and CBF were compared between different labeling schemes.

### 3.4 Statistical analysis

The mean tissue signal (BS level), normalized ASL signal, tSNR, and CBF in the ROIs across subjects were tested for normality (Jarque‐Bera test). All values were normally distributed except the tissue signals acquired using PASL, and Wilcoxon signed rank test was used when needed. ASL signal, tSNR, and CBF in the GM and WM ROIs were compared using 1‐way analysis of variance (with Tukey–Kramer adjustment) and multiple pairwise *t* tests. Significant differences were identified with *P* < 0.05 (uncorrected). Bonferroni correction was applied on the threshold when multiple pairwise comparisons (reported as n) were performed, and uncorrected *P* value are reported.

## 4 RESULTS

### 4.1 SNR efficiency optimization

**FIGURE 3:**
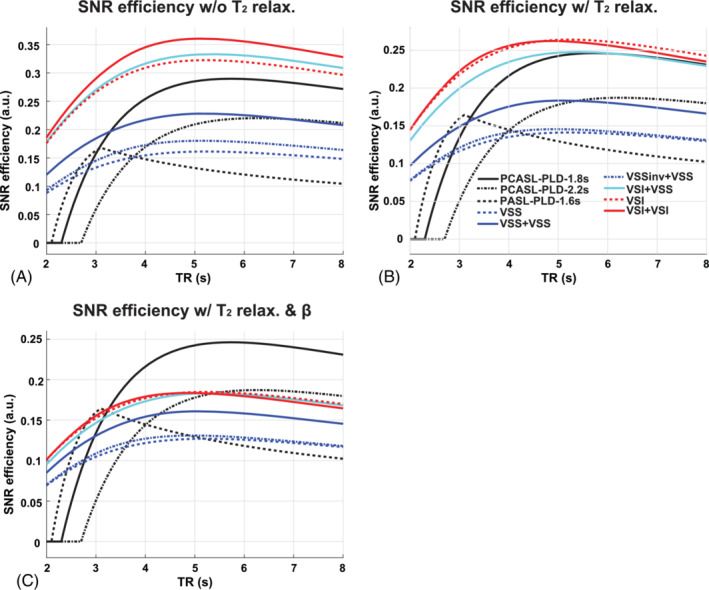
SNR efficiency (SNR per unit time) comparison between PASL, PCASL, and VSASL with different labeling schemes under different scenarios: (A) all the ASL schemes have an ideal labeling efficiency of 1; (B) $\alpha_{\text{PASL}}=0.98$, $\alpha_{\text{PCASL}}=0.85$, for VSASL schemes, T_2_ relaxation during the 2 VS modules were included and $\beta_{\mathrm{VSS}}=\beta_{\mathrm{VSI}}$ = 1; (C) scenario (B) with $\beta_{\mathrm{VSS}}$ = 0.9 and $\beta_{\mathrm{VSI}}$ = 0.7. PASL, pulsed ASL; PCASL, pseudo‐continuous ASL; VSASL, velocity‐selective ASL.

The results of SNR efficiency simulation are shown in Figure 3. The SNR efficiencies of VSASL methods generally peaked around TR = 5 s. Under ideal conditions, that is, the labeling efficiencies were 1 for all methods, the maximal SNR efficiency of dm‐VSASL (TI_1/2_ = 1.52/0.48 s) was increased by 12.0% compared to sm‐VSASL (TI = 1.4 s) for both VSS and VSI. For dm‐VSS, the increase was smaller than that of mm‐VSS (TI_1/2_ = 1.18/0.82 s, 41.6%). Note the SNR efficiency advantage of VSASL methods compared to PCASL and PASL in this ideal scenario. When T_2_ relaxation was included (β = 1), the maximal SNR efficiency of dm‐VSI (TI_1/2_ = 1.46/0.54 s) was almost the same as VSI (TI = 1.4 s) labeling; dm‐VSS had a smaller increase (3.4%), whereas mm‐VSS (TI_1/2_ = 1.16/0.84 s) still had an increase of 30.2%, compared to VSS labeling. When realistic VS pulses were considered (with T_2_ relaxation and β < 1), the SNR efficiencies of VSASL methods would further decrease. Compared to VSI (TI = 1.4 s), dm‐VSI (TI_1/2_ = 1.46/0.54 s) still had a comparable SNR efficiency (0.4% less), and the hybrid dm‐VSASL (VSI + VSS, TI_1/2_ = 1.43/0.57 s) had a slightly lower SNR efficiency (2.7% less). Compared to VSS, dm‐VSS (TI_1/2_ = 1.48/0.52 s) had a slightly higher SNR efficiency (3.4% more), and mm‐VSS (TI_1/2_ = 1.18/0.82 s) had an increase of 28.5%. Note that under this realistic scenario, PCASL had a higher SNR efficiency than VSASL methods when PLD was not long (1.8 s); when a longer PLD (e.g., 2.2 s) was used in PCASL due to prolonged ATTs, dm‐VSASL using at least 1 VSI module would have a comparable SNR efficiency and would be more SNR‐efficient if the PLD in PCASL had to be further increased.

### 4.2 In vivo results

**FIGURE 4:**
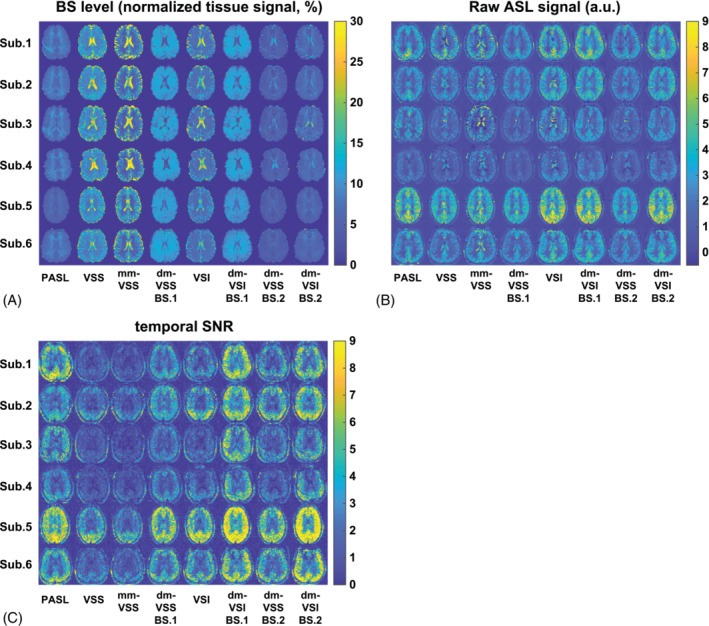
(A) BS level; (B) raw ASL signal; and (C) temporal SNR (tSNR) maps across subjects. Note the effective suppression of CSF signals, reduced artifacts, and improved tSNR using the proposed dm‐VSASL methods. BS, background suppression; tSNR, temporal SNR.

The BS level maps are shown in Figure 4A. The averaged BS levels in different tissue ROIs and across subjects are summarized in Table 1. Different and consistent BS levels were achieved using different ASL methods across subjects. In VSS, mm‐VSS, and VSI, the CSF signal could not be sufficiently suppressed (≥ 15.7%) despite the effort in BS timing optimization. In contrast, the new dm‐VSASL strategy achieved sufficient suppression across all brain tissues in both BS1 and BS2, including excellent suppression of CSF compared to VSS, mm‐VSS, and VSI (≤ 7.1%, *P* < 0.001, *n* = 3).

**TABLE 1:** Normalized BS levels in GM, WM, and CSF; and the averaged tSNR in GM and WM across subjects

ASL schemes (*n* = 6)		PASL	VSS	mm‐VSS	dm‐VSS (BS1)	VSI	dm‐VSI (BS1)	dm‐VSS (BS2)	dm‐VSI (BS2)
Mean BS level (mean ± SEM, %)	**GM**	5.8 ± 0.5	13.4 ± 0.9	15.7 ± 2.2	9.8 ± 0.4	10.6 ± 1.3	9.1 ± 0.2	4.7 ± 0.4	5.7 ± 0.5
	**WM**	8.5 ± 0.7	8.6 ± 0.2	5.1 ± 0.2	11.4 ± 0.3	6.7 ± 0.3	11.5 ± 0.4	5.2 ± 0.2	7.1 ± 0.2
	**CSF**	5.4 ± 0.4	20.4 ± 2.9	23.7 ± 3.7	5.2 ± 1.3	15.7 ± 2.1	5.5 ± 0.9	7.1 ± 0.8	6.3 ± 0.5
Mean tSNR (mean ± SEM)	**GM**	4.7 ± 1.3	2.1 ± 1.0	1.8 ± 0.7	4.0 ± 1.1	3.2 ± 1.4	6.2 ± 1.2	3.7 ± 1.4	5.6 ± 2.0
	**WM**	1.9 ± 0.6	0.9 ± 0.2	0.8 ± 0.2	1.3 ± 0.3	1.5 ± 0.5	2.3 ± 0.4	1.3 ± 0.4	2.2 ± 0.5

*Note*: dm‐VSS (BS1) and dm‐VSI (BS1) were referred to as dm‐VSS and dm‐VSI for simplicity in later analyses.

Abbreviations: ASL, arterial spin labeling; BS, background suppression; dm‐VSI, dual‐module velocity‐selective inversion; dm‐VSS, dual‐module velocity‐selective saturation; GM, gray matter; mm‐VSS, multi‐module velocity‐selective saturation; PASL, pulsed ASL; SEM, standard error of the mean; tSNR, temporal SNR; VSS, velocity‐selective saturation; VSI, velocity‐selective inversion; WM, white matter.

Of the 2 BS settings using dm‐VSS and dm‐VSI, BS1 had higher GM (*P* < 0.0006) and WM (*P* < 1.3 × 10^−6^) tissue signal than BS2 but lower CSF tissue signal (though not significant, *P* = 0.11 and 0.17 for dm‐VSS and dm‐VSI, respectively). BS1 produced ASL images with higher quality than BS2, that is, with higher (though not significantly) tSNR (*P* = 0.22 for dm‐VSS, and *P* = 0.27 for dm‐VSI, respectively), and should provide a reasonable representation of the performance of dm‐VSASL. Therefore, further analyses focused on the measurements using the BS1 setting for comparisons between different labeling schemes. For simplicity, dm‐VSS with BS1 was referred to as dm‐VSS and dm‐VSI with BS1 as dm‐VSI unless specified.

Raw ASL signal maps are shown in Figure 4B. CSF artifacts were noticeable using VSS, mm‐VSS, and VSI; whereas dm‐VSASL methods yielded markedly improved quality with almost no CSF artifact, especially around the ventricles. These observations were confirmed by the tSNR maps shown in Figure 4C, where obvious tSNR improvement was observed in both the cortical GM and the deep GM regions when comparing dm‐VSS versus VSS and mm‐VSS, and dm‐VSI versus VSI. Note that there was some regional ASL signal reduction in the frontal area in subjects 4 and 6 with VSI‐based labeling but not with VSS‐based labeling, likely due to its sensitivity to field inhomogeneities (see discussion).

Significantly improved temporal stability using dm‐VSASL schemes can be appreciated in an example of the raw ASL signal time series shown in Supporting Information Figure S2. High signal fluctuations were observed in regions where CSF signals were not sufficiently suppressed with VSS, mm‐VSS, and VSI labeling. Even though some of the fluctuations were averaged out, there were erroneous ASL signals in voxels with dominant CSF signals, for example, around ventricles and sulci. In contrast, both dm‐VSS and dm‐VSI produced ASL signals with high temporal stability throughout the brain and had a better performance than PASL.

**FIGURE 5:**
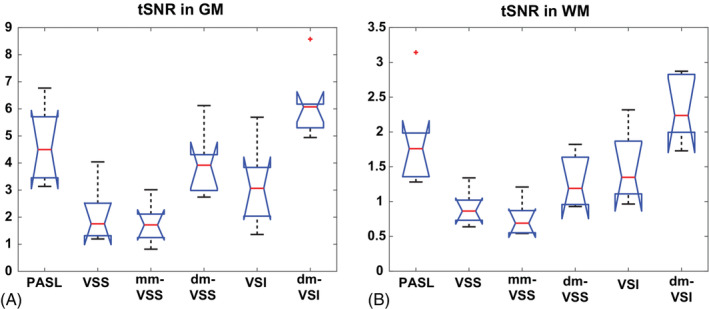
Boxplots of the averaged tSNR in the (A) and WM (B) ROIs across subjects. GM, gray matter; ROI, region of interest; tSNR, temporal SNR; WM, white matter.

Averaged tSNR in GM and WM ROIs across subjects is shown in the boxplots in Figure 5A,B, respectively, and summarized in Table 1. Compared to their single‐module counterparts, dm‐VSS significantly improved the tSNR by 90.8% in GM (*P* = 1.9 × 10^−5^) and 41.5% in WM (*P* = 1.8 × 10^−3^); and dm‐VSI improved the tSNR by 94.9% in GM (*P* = 1.4 × 10^−4^) and 55.1% in WM (*P* = 8.1 × 10^−4^). Dm‐VSS had a significantly higher tSNR in GM (25.9% higher, *P* = 0.0083) but a lower tSNR in WM (13.8% lower, *P* = 0.036) than VSI. Compared to VSS, mm‐VSS had a similar tSNR in GM (*P* = 0.11) but a lower tSNR in WM (*P* = 4.5 × 10^−4^). In GM, dm‐VSI had the highest tSNR among all methods (*P* < 0.006, *n* = 8); in WM, dm‐VSI had the highest tSNR among the VSASL methods (*P* < 8.1 × 10^−4^, *n* = 8) and tended to have a higher tSNR than PASL (*P* = 0.048, not significantly after correction, *n* = 8).

**FIGURE 6:**
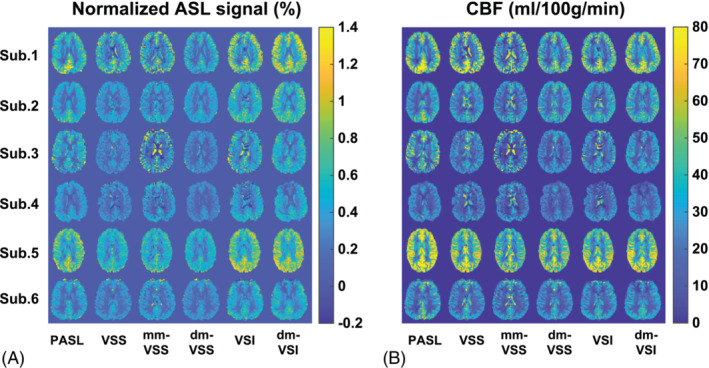
(A) The normalized ASL signal maps (with respect to the reference image) and (B) corresponding quantitative CBF maps from a representative slice across subjects. ASL, arterial spin labeling; CBF, cerebral blood flow.

**FIGURE 7:**
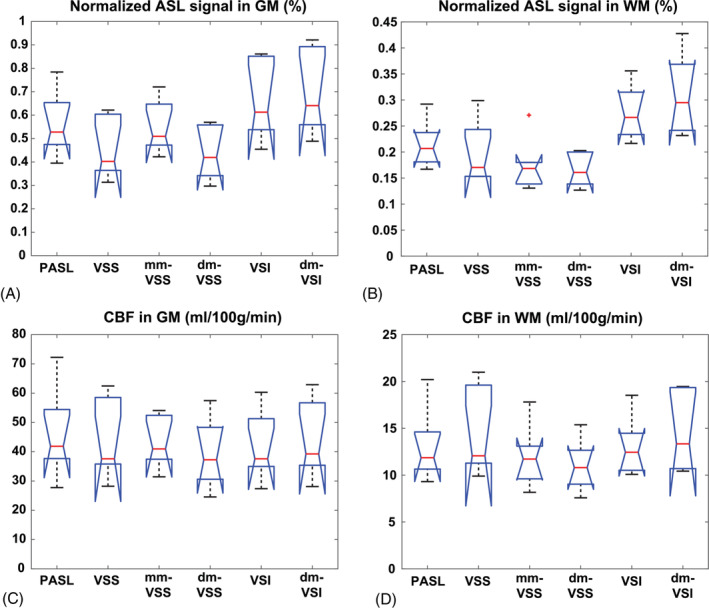
Boxplots of the normalized ASL signal (A and B), and the CBF (C and D) in the GM and WM ROIs, averaged across subjects

Normalized ASL signal and quantified CBF maps are shown in Figure 6. The averaged values in GM and WM ROIs across subjects are shown in Figure 7 and summarized in Table 2. For GM ASL signal, dm‐VSI increased the ASL signal by 5.4% (*P* = 0.018) compared to VSI labeling; mm‐VSS increased ASL signal by 21.1% (*P* = 0.0009) compared to VSS, consistent with previous results^14^, ^22^; whereas dm‐VSS had a comparable ASL signal compared to VSS (*P* = 0.27). Compared to VSS, VSI and dm‐VSI increased the ASL signal by 45.1% (*P* = 0.0002, *n* = 4) and 53.0% (*P* = 0.0001, *n* = 4), respectively. For WM ASL signal, compared to VSS, mm‐VSS (*P* = 0.33) and dm‐VSS (*P* = 0.15) had similar ASL signal, VSI and dm‐VSI increased the ASL signal by 39.1% (*P* = 0.012, *n* = 4) and 56.3% (*P* = 0.0006, *n* = 4), respectively. Compared to VSI, dm‐VSI yielded 12.4% higher signal in WM, though not significantly (*P* = 0.15).

**TABLE 2:** Averaged normalized ASL signal and CBF in GM and WM ROIs across subjects, and averaged gray/white ratios

ASL schemes (*n* = 6)		PASL	VSS	mm‐VSS	dm‐VSS	VSI	dm‐VSI
Normalized ASL signal (mean ± SEM, %)	**GM**	0.56 ± 0.13	0.45 ± 0.12	0.55 ± 0.10	0.43 ± 0.11	0.66 ± 0.15	0.69 ± 0.17
	**WM**	0.22 ± 0.04	0.20 ± 0.05	0.18 ± 0.05	0.16 ± 0.03	0.28 ± 0.05	0.31 ± 0.07
CBF (mean ± SEM, ml/100 g/min)	**GM**	46.0 ± 14.2	43.4 ± 12.6	42.9 ± 8.1	39.2 ± 11.0	41.5 ± 11.0	43.6 ± 12.3
	**WM**	13.1 ± 3.6	14.3 ± 4.3	12.0 ± 3.1	11.0 ± 2.6	13.1 ± 3.0	14.4 ± 3.9
Gray/white ratio		3.51	3.03	3.57	3.55	3.17	3.02

*Note*: The signal attenuation from additional background suppression pulses was corrected in ASL signal and CBF calculation.

Abbreviations: CBF, cerebral blood flow; ROI, region of interest.

There was no significant difference in CBF measured using different ASL methods in GM (*P* = 0.97) or WM (*P* = 0.62) ROIs according to 1‐way analysis of variance. Averaged gray/white ratios were within the range of 3.02 ˜ 3.57. These values are reported in Table 2.

## 5 DISCUSSION

**FIGURE 8:**
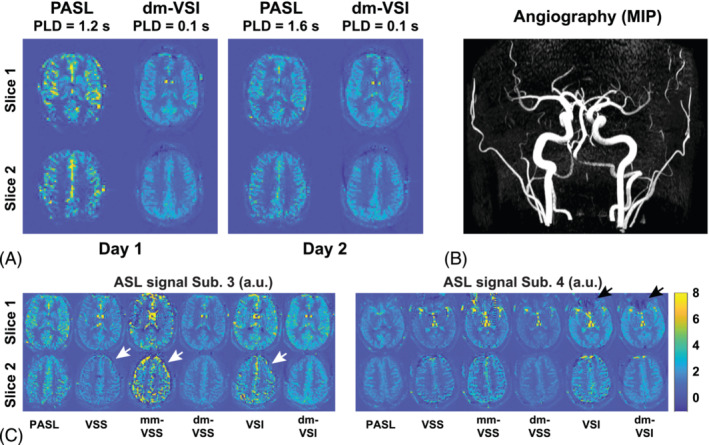
(A) Examples showing the ATT artifacts (delayed flow and strong intravascular signals are indicated by white and black arrows, respectively) using PASL with a normal PLD of 1.2 s in subject 3; (B) the MIP of the angiogram from subject 3 showing a tortuous vertebrobasilar artery, likely to be the cause of the ATT artifacts; (C) examples of CSF artifacts (left panel, white arrows) using single‐module VSASL (VSS and VSI) and previous multi‐module VSASL (mm‐VSS); and artifacts using the VSI pulses (right panel, black arrows). ATT, arterial transit time; MIP, maximum‐intensity‐project.

The novel dm‐VSASL strategy offers a few distinctive advantages compared to existing VSASL methods: (1) the label/control condition switching in the second VS module creates a more balanced distribution of VS (motion‐sensitizing) gradients and diffusion weighting in the label/control acquisition, reducing artifacts and errors from sources such as diffusion attenuation, ECs, and possibly pulsatile motion such as in CSF; (2) the inversion effect from the first VS module at an early time point enables more flexible and effective BS, especially of CSF, resulting in further noise reduction; (3) dual‐module labeling can increase the SNR efficiency, improving the quality and/or the efficiency of VSASL scans. These features significantly enhanced the accuracy and the robustness of VSASL. Combined with its insensitivity to ATT artifacts and SNR advantage in presence of delayed blood flow, VSASL is particularly suited for perfusion imaging applications such as in vascular disease cohorts or in aging population. VSASL's insensitivity to ATT effects has been demonstrated in healthy subjects and patients.^14^, ^33^, ^34^ An interesting case was encountered in this study and is shown in Figure 8A,B, where ATT artifacts were accidentally observed in a young healthy subject using PASL with a PLD of 1.2 s (corresponding to a TI of 2 s, already longer than recommended TI value of 1.8 s), and were mostly gone (ASL images still a little grainy) after increasing the PLD to 1.6 s; whereas VSASL (only dm‐VSI is shown) yielded consistent and ATT–artifact‐free perfusion maps.

In principle, dm‐VSASL is applicable with any even number of VS modules (e.g., more than 2); however, as each additional VS labeling module results in higher signal reduction due to imperfect labeling, it may not be beneficial to use more than 2 VS module in practice. The SNR efficiency simulation results also emphasized the need for robust VS modules of higher β and shorter effect TE.

The constraint of ${\mathrm{TI}}_{1}+{\mathrm{TI}}_{2}<{\mathrm{BD}}_{\max}=2\;s$ was adopted for accurate quantification of VSASL in the brain, based on estimation from a few healthy subjects with a whole‐body RF coil for labeling.^14^ ${\mathrm{BD}}_{\max}$ may vary in different situations, such as applications in different organs, using different RF coils or in subjects with abnormal arterial velocities. If ${\mathrm{BD}}_{\max}$ is smaller, the timing optimization for optimal SNR efficiency should be adjusted accordingly; if ${\mathrm{BD}}_{\max}$ is larger, for example, in vascular disease patients with slow and delayed flow, the constraint remains valid and the timings reported in this study are directly applicable, with a slightly suboptimal SNR efficiency. For example, if ${\mathrm{BD}}_{\max}$ increases to 2.5 s, using the timings derived under ${\mathrm{BD}}_{\max}=2\;s$ will still achieve 95% of the optimal SNR efficiency.

Compared to PASL, VSASL methods yielded comparable CBF values, suggesting the β values used in this study were reasonable. This is also consistent with the results from a study performed on a different scanner and using PCASL as the reference.^22^ Compared to VSI, dm‐VSI yielded a 5.4% increase of GM ASL signal in in vivo experiments despite the possibility of VSI having artificially higher signal due to diffusion attenuation effect from CSF. This is higher than predicted, possibly due to an improved overall labeling robustness using the dual‐module strategy, that is, a slightly higher averaged β in dual‐module labeling (e.g., an improved β in the second VS module) than in single‐module labeling. On the other hand, the ASL signal improvement of dm‐VSS with respect to VSS was lower than that predicted by simulation, suggesting either reduced averaged β in dm‐VSS labeling or more severe CSF contamination in VSS labeling. The latter is more likely, judging from the ASL signal maps compared to PASL and the fact that the β of VSS is already close to 1. Nevertheless, accurate measurement of the labeling efficiencies (especially β) of different VS modules and under different labeling strategies is needed for further improved quantification accuracy using VSASL.

Dm‐VSASL with BS1 (higher GM/WM and lower CSF signals) had a better tSNR performance than with BS2 (lower GM/WM but slightly higher CSF signals), indicating that CSF contributed much higher noise than GM or WM. This is also supported by the observation that the tSNR improvement with dm‐VSASL in GM is higher than that in WM (90.8% vs. 41.5% with VSS and 94.9% vs. 55.1% with VSI) compared with sm‐VSASL, likely due to a generally higher partial volume of CSF in “GM” voxels. In PASL and PCASL, the labeling and imaging volumes are separated, and the labeling mechanisms hardly interact with CSF. Therefore, CSF is typically not a significant source of signal variations in PASL and PCASL and can be well suppressed using existing methods.^24^, ^35^ In contrast, the labeling in VSASL is global and interacts with tissues in the imaging volume in a more complex way. In sm‐VSASL and mm‐VSASL, effective suppression of CSF is difficult and limited by the timing constraints for optimizing the SNR, resulting in insufficient BS levels (typically >20%) and high noise from CSF. In addition, the diffusion attenuation effect from CSF is also a greater source of error than that from GM and WM in VSASL. Therefore, good suppression of CSF should be prioritized in VSASL (additional examples of CSF artifacts can be found in Figure 8C). Previous studies had emphasized its importance with efforts to directly suppress CSF signal at the cost of SNR^36^ or to correct for its erroneous signal with additional post‐processing steps.^14^ With the new dm‐VSASL schemes, the BS optimization in VSASL, especially for CSF, is much more amiable. For example, both sm‐VSS and dm‐VSS had the same number (2) of effective BS pulses, including 1 from the built‐in inversion in VSS_inv_, and dm‐VSS had better BS and tSNR performance. The 2 sets of BS parameters used in this study were for demonstration of the flexibility and effectiveness of BS with dm‐VSASL; its optimization was not a focus and should be investigated further. In general, more additional BS pulses may provide more flexibility in BS, but the associated signal reduction and increased specific absorption rate should also be taken into consideration in pulse sequence design. In addition, complex reconstruction would be beneficial as it allows more aggressive timings for improved BS while avoiding rectification errors.

Aside from the effective suppression of CSF, the more balanced VS gradient application in label/control images also contributed significantly to the tSNR improvement with dm‐VSASL. This is more evident when comparing the tSNR in WM, where the partial volume fraction of CSF is much smaller than in GM. The tSNR increased by 41.5% (dm‐VSS vs. VSS) and 55.1% (dm‐VSI vs. VSI) despite higher tissue signals (less BS) in WM with dm‐VSASL than with sm‐VSASL.

In addition to the application in baseline perfusion measurement, VSASL is useful in fMRI studies for its insensitivity to ATT effects.^37^ And a recent study demonstrated the SNR advantage of VSI‐based VSASL in fMRI.^23^ With almost doubled tSNR in GM compared to existing VSASL methods, dm‐VSI should be an excellent tool for imaging functional changes of blood flow.

Despite the excellent SNR of VSI‐based labeling, its current implementations (rect‐VSI and sinc‐VSI) are still somewhat susceptible to field inhomogeneities as predicted by Bloch simulation and shown in vivo.^20^, ^22^ For example, as shown in Figure 8C, insufficient labeling and artifacts were observed in regions with compromised homogeneity in B_0_ and B_1_ fields. Improving the robustness to field inhomogeneities is highly desired for VSI‐based labeling and should be studied further. In addition, better shimming should also help improve the performance. In contrast, sBIR8 VSS‐based labeling did not generate such artifacts, suggesting dm‐VSS may be more suitable for applications with higher field inhomogeneities. Dual‐module labeling increases the specific absorption rate compared to single‐module labeling. Though dm‐VSI has slightly higher specific absorption rate than dm‐VSS, both were within the normal safety limit and can be reduced with pulse optimization.

## 6 CONCLUSION

The dm‐VSASL strategy can significantly reduce noise and artifacts that are typically encountered with existing VSASL methods, offering dramatically enhanced tSNR in both GM and WM. It is achieved by utilizing a more balanced VS gradient configuration in control and label image acquisition and enabling more efficient suppression of background tissue signals, especially of CSF. A slight SNR improvement is also achieved with dm‐VSI compared to VSI. With enhanced labeling robustness and reduced artifacts, dm‐VSASL can measure perfusion more reliably and accurately, especially in applications where ATT effects are concerned.

## FUNDING INFORMATION

This work is supported by the National Institutes of Health (NIH), grant R01EB033210.

## Supporting information


**FIGURE S1:** Dm‐VSASL signal maps without and with the control/label condition switching in the second VS module. Left panel: the first VS module is symmetric BIR8 (sBIR8) based VSS_inv_ and the second VS module was sBIR8 VSS; right panel: both VS modules were sinc‐VSI. Without the control/label condition switching, the dm‐VSASL signals from the two VS modules had opposite signs and almost canceled each other.
**FIGURE S2**: Representative raw ASL signal time series from Subject 2 (only the first 11 time points are shown for PASL), the acquisition time for each image was 16 s for PASL and 20 s for VSASL. Note the superior stability of labeling using dm‐VSS and dm‐VSI across time.

## References

[1] Wong EC , Cronin M , Wu W‐C , Inglis B , Frank LR , Liu TT . Velocity‐selective arterial spin labeling. Magn Reson Med. 2006;55:1334‐1341. 16700025 10.1002/mrm.20906

[2] Detre JA , Leigh JS , Williams DS , Koretsky AP . Perfusion imaging. Magn Reson Med. 1992;23:37‐45. 1734182 10.1002/mrm.1910230106

[3] Williams DS , Detre JA , Leigh JS , Koretsky AP . Magnetic resonance imaging of perfusion using spin inversion of arterial water. Proc Natl Acad Sci U S A. 1992;89:212‐216. 1729691 10.1073/pnas.89.1.212 PMC48206

[4] Alsop DC , Detre JA . Reduced transit‐time sensitivity in noninvasive magnetic resonance imaging of human cerebral blood flow. J Cereb Blood Flow Metab. 1996;16:1236‐1249. 8898697 10.1097/00004647-199611000-00019

[5] Gonzalez‐At JB , Alsop DC , Detre JA . Cerebral perfusion and arterial transit time changes during task activation determined with continuous arterial spin labeling. Magn Reson Med. 2000;43:739‐746. 10800040 10.1002/(sici)1522-2594(200005)43:5<739::aid-mrm17>3.0.co;2-2

[6] van Osch MJ , Hendrikse J , van der Grond J . Sensitivity comparison of multiple vs. single inversion time pulsed arterial spin labeling fMRI. J Magn Reson Imaging. 2007;25:215‐221. 17154371 10.1002/jmri.20823

[7] Zhou J , van Zijl PC . Effect of transit times on quantification of cerebral blood flow by the FAIR T(1)‐difference approach. Magn Reson Med. 1999;42:890‐894. 10542347 10.1002/(sici)1522-2594(199911)42:5<890::aid-mrm8>3.0.co;2-8

[8] Edelman RR , Siewert B , Darby DG , et al. Qualitative mapping of cerebral blood flow and functional localization with echo‐planar MR imaging and signal targeting with alternating radio frequency. Radiology. 1994;192:513‐520. 8029425 10.1148/radiology.192.2.8029425

[9] Kim SG . Quantification of relative cerebral blood flow change by flow‐sensitive alternating inversion recovery (FAIR) technique: application to functional mapping. Magn Reson Med. 1995;34:293‐301. 7500865 10.1002/mrm.1910340303

[10] Wong EC , Buxton RB , Frank LR . Implementation of quantitative perfusion imaging techniques for functional brain mapping using pulsed arterial spin labeling. NMR Biomed. 1997;10:237‐249. 9430354 10.1002/(sici)1099-1492(199706/08)10:4/5<237::aid-nbm475>3.0.co;2-x

[11] Golay X , Stuber M , Pruessmann KP , Meier D , Boesiger P . Transfer insensitive labeling technique (TILT): application to multislice functional perfusion imaging. J Magn Reson Imaging. 1999;9:454‐461. 10194717 10.1002/(sici)1522-2586(199903)9:3<454::aid-jmri14>3.0.co;2-b

[12] Dai WY , Garcia D , de Bazelaire C , Alsop DC . Continuous flow‐driven inversion for arterial spin labeling using pulsed radio frequency and gradient fields. Magn Reson Med. 2008;60:1488‐1497. 19025913 10.1002/mrm.21790 PMC2750002

[13] Kwong KK , Belliveau JW , Chesler DA , et al. Dynamic magnetic resonance imaging of human brain activity during primary sensory stimulation. Proc Natl Acad Sci U S A. 1992;89:5675‐5679. 1608978 10.1073/pnas.89.12.5675 PMC49355

[14] Guo J , Wong EC . Increased SNR efficiency in velocity selective arterial spin labeling using multiple velocity selective saturation modules (mm‐VSASL). Magn Reson Med. 2015;74:694‐705. 25251933 10.1002/mrm.25462 PMC4369468

[15] Qin Q , Alsop DC , Bolar DS , et al. Velocity‐selective arterial spin labeling perfusion MRI: a review of the state of the art and recommendations for clinical implementation. Magn Reson Med. 2022;88:1528‐1547. 35819184 10.1002/mrm.29371 PMC9543181

[16] Guo J , Meakin JA , Jezzard P , Wong EC . An optimized design to reduce eddy current sensitivity in velocity‐selective arterial spin labeling using symmetric BIR‐8 pulses. Magn Reson Med. 2015;73:1085‐1094. 24710761 10.1002/mrm.25227

[17] Meakin JA , Jezzard P . An optimized velocity selective arterial spin labeling module with reduced eddy current sensitivity for improved perfusion quantification. Magn Reson Med. 2013;69:832‐838. 22556043 10.1002/mrm.24302

[18] Norris DG , Schwartzbauer C . Velocity selective radiofrequency pulse trains. J Magn Reson. 1999;137:231‐236. 10053152 10.1006/jmre.1998.1690

[19] de Rochefort L , Maitre X , Bittoun J , Durand E . Velocity‐selective RF pulses in MRI. Magn Reson Med. 2006;55:171‐176. 16342055 10.1002/mrm.20751

[20] Qin Q , van Zijl PC . Velocity‐selective‐inversion prepared arterial spin labeling. Magn Reson Med. 2016;76:1136‐1148. 26507471 10.1002/mrm.26010 PMC4848210

[21] Alsop DC , Detre JA , Golay X , et al. Recommended implementation of arterial spin‐labeled perfusion MRI for clinical applications: a consensus of the ISMRM perfusion study group and the European consortium for ASL in dementia. Magn Reson Med. 2015;73:102‐116. 24715426 10.1002/mrm.25197 PMC4190138

[22] Guo J , Das S , Hernandez‐Garcia L . Comparison of velocity‐selective arterial spin labeling schemes. Magn Reson Med. 2021;85:2027‐2039. 33128484 10.1002/mrm.28572 PMC11155614

[23] Hernandez‐Garcia L , Nielsen JF , Noll DC . Improved sensitivity and temporal resolution in perfusion FMRI using velocity selective inversion ASL. Magn Reson Med. 2019;81:1004‐1015. 30187951 10.1002/mrm.27461 PMC6289627

[24] Ye FQ , Frank JA , Weinberger DR , McLaughlin AC . Noise reduction in 3D perfusion imaging by attenuating the static signal in arterial spin tagging (ASSIST). Magn Reson Med. 2000;44:92‐100. 10893526 10.1002/1522-2594(200007)44:1<92::aid-mrm14>3.0.co;2-m

[25] Guo J , Wong EC . Venous oxygenation mapping using velocity‐selective excitation and arterial nulling. Magn Reson Med. 2012;68:1458‐1471. 22294414 10.1002/mrm.24145 PMC3342455

[26] Buxton RB , Frank LR , Wong EC , Siewert B , Warach S , Edelman RR . A general kinetic model for quantitative perfusion imaging with arterial spin labeling. Magn Reson Med. 1998;40:383‐396. 9727941 10.1002/mrm.1910400308

[27] Wong EC , Guo J . BIR‐4 based B1 and B0 insensitive velocity selective pulse trains. In Proc. Intl. Soc. Mag. Reson. Med. 2010:2853.

[28] Jung Y , Wong EC , Liu TT . Multiphase pseudocontinuous arterial spin labeling (MP‐PCASL) for robust quantification of cerebral blood flow. Magn Reson Med. 2010;64:799‐810. 20578056 10.1002/mrm.22465

[29] Kwong KK , Chesler DA , Weisskoff RM , et al. MR perfusion studies with T1‐weighted echo planar imaging. Magn Reson Med. 1995;34:878‐887. 8598815 10.1002/mrm.1910340613

[30] Luh WM , Wong EC , Bandettini PA , Hyde JS . QUIPSS II with thin‐slice TI1 periodic saturation: a method for improving accuracy of quantitative perfusion imaging using pulsed arterial spin labeling. Magn Reson Med. 1999;41:1246‐1254. 10371458 10.1002/(sici)1522-2594(199906)41:6<1246::aid-mrm22>3.0.co;2-n

[31] Murphy K , Bodurka J , Bandettini PA . How long to scan? The relationship between fMRI temporal signal to noise ratio and necessary scan duration. Neuroimage. 2007;34:565‐574. 17126038 10.1016/j.neuroimage.2006.09.032 PMC2223273

[32] Smith SM , Jenkinson M , Woolrich MW , et al. Advances in functional and structural MR image analysis and implementation as FSL. Neuroimage. 2004;23:S208‐S219. 15501092 10.1016/j.neuroimage.2004.07.051

[33] Guo J , Fan A , Lebel MR , Holdsworth S , Shankaranarayanan A , Zaharchuk G . Extreme ASL: challenges and solutions to improve perfusion imaging in patients with markedly prolonged arterial transit delays. In Proc. Intl. Soc. Mag. Reson. Med. 2017:683.

[34] Bolar DS , Gagoski B , Orbach DB , et al. Comparison of CBF measured with combined velocity‐selective arterial spin‐labeling and pulsed arterial spin‐labeling to blood flow patterns assessed by conventional angiography in pediatric Moyamoya. AJNR Am J Neuroradiol. 2019;40:1842‐1849. 31694821 10.3174/ajnr.A6262 PMC6975103

[35] Maleki N , Dai W , Alsop DC . Optimization of background suppression for arterial spin labeling perfusion imaging. MAGMA. 2012;25:127‐133. 22009131 10.1007/s10334-011-0286-3 PMC3671066

[36] Wong EC , Liu TT , Luh WM , Frank LR , Buxton RB . T(1) and T(2) selective method for improved SNR in CSF‐attenuated imaging: T(2)‐FLAIR. Magn Reson Med. 2001;45:529‐532. 11241715 10.1002/1522-2594(200103)45:3<529::aid-mrm1071>3.0.co;2-l

[37] Wu WC , Wong EC . Feasibility of velocity selective arterial spin labeling in functional MRI. J Cereb Blood Flow Metab. 2007;27:831‐838. 16926843 10.1038/sj.jcbfm.9600386

