# Supplementary material for: Robust dual‐module velocity‐selective arterial spin labeling (dm‐VSASL) with velocity‐selective saturation and inversion
Source: Magn Reson Med. 2022 Nov 6;89(3):1026–40. doi: 10.1002/mrm.29513 (PMC9792445; doi:10.1002/mrm.29513)
Supplement: Supplementary file 1 — FIGURE S1: Dm‐VSASL signal maps without and with the control/label condition switching in the second VS module. Left panel: the first VS module is symmetric BIR8 (sBIR8) based VSSinv and the second VS module was sBIR8 VSS; right panel: both VS modules were sinc‐VSI. Without the control/label condition switching, the dm‐VSASL signals from the two VS modules had opposite signs and almost canceled each other. FIGURE S2: Representative raw ASL signal time series from Subject 2 (only the first 11 time points are shown for PASL), the acquisition time for each image was 16 s for PASL and 20 s for VSASL. Note the superior stability of labeling using dm‐VSS and dm‐VSI across time. [file MRM-89-1026-s001.docx]

**Supporting Inforamtion**


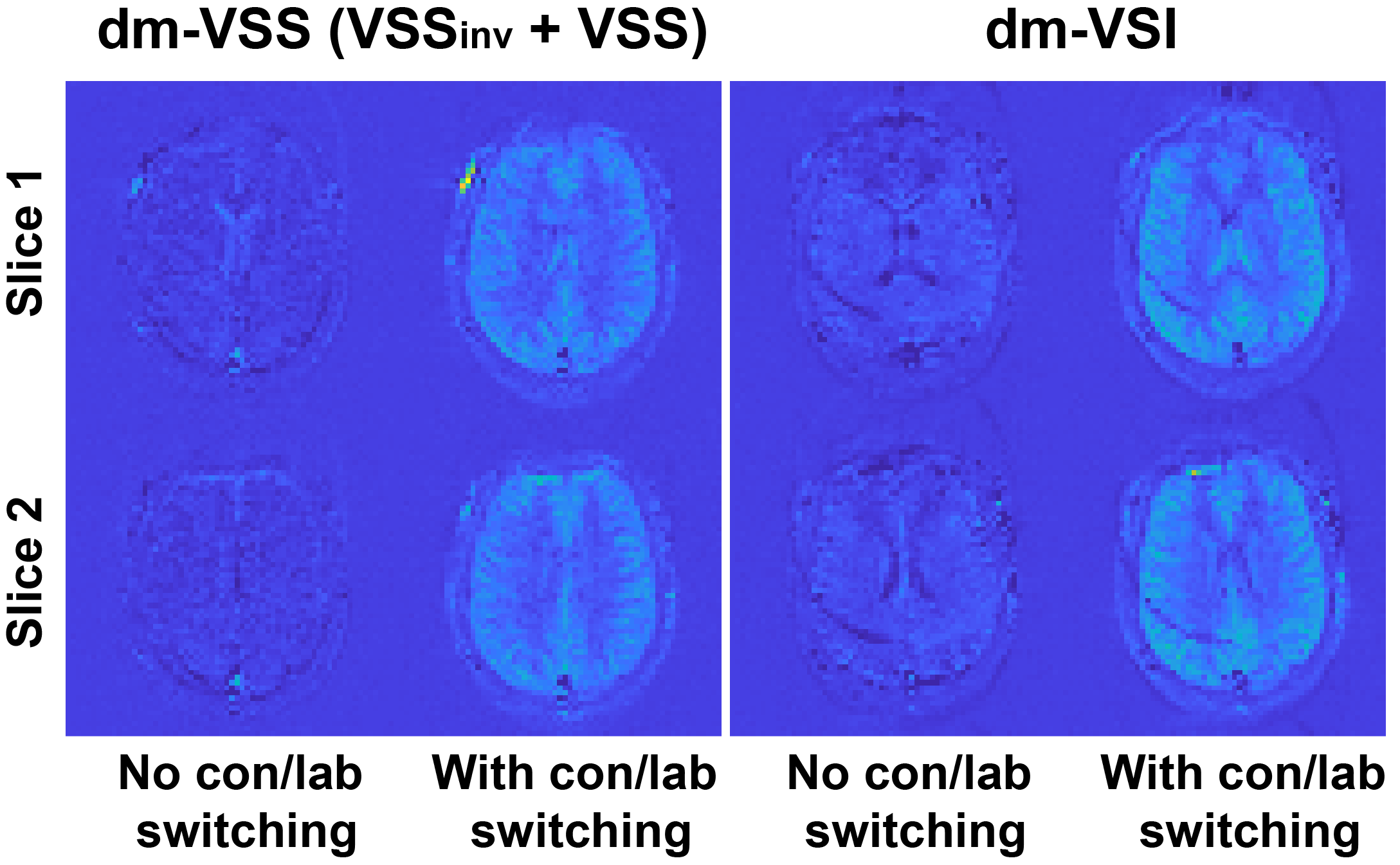


**Supporting Information Figure S1**: Dm-VSASL signal maps without and with the control/label condition switching in the second VS module. Left panel: the first VS module is symmetric BIR8 (sBIR8) based VSS_inv_ and the second VS module was sBIR8 VSS; right panel: both VS modules were sinc-VSI. Without the control/label condition switching, the dm-VSASL signals from the two VS modules had opposite signs and almost canceled each other.

**
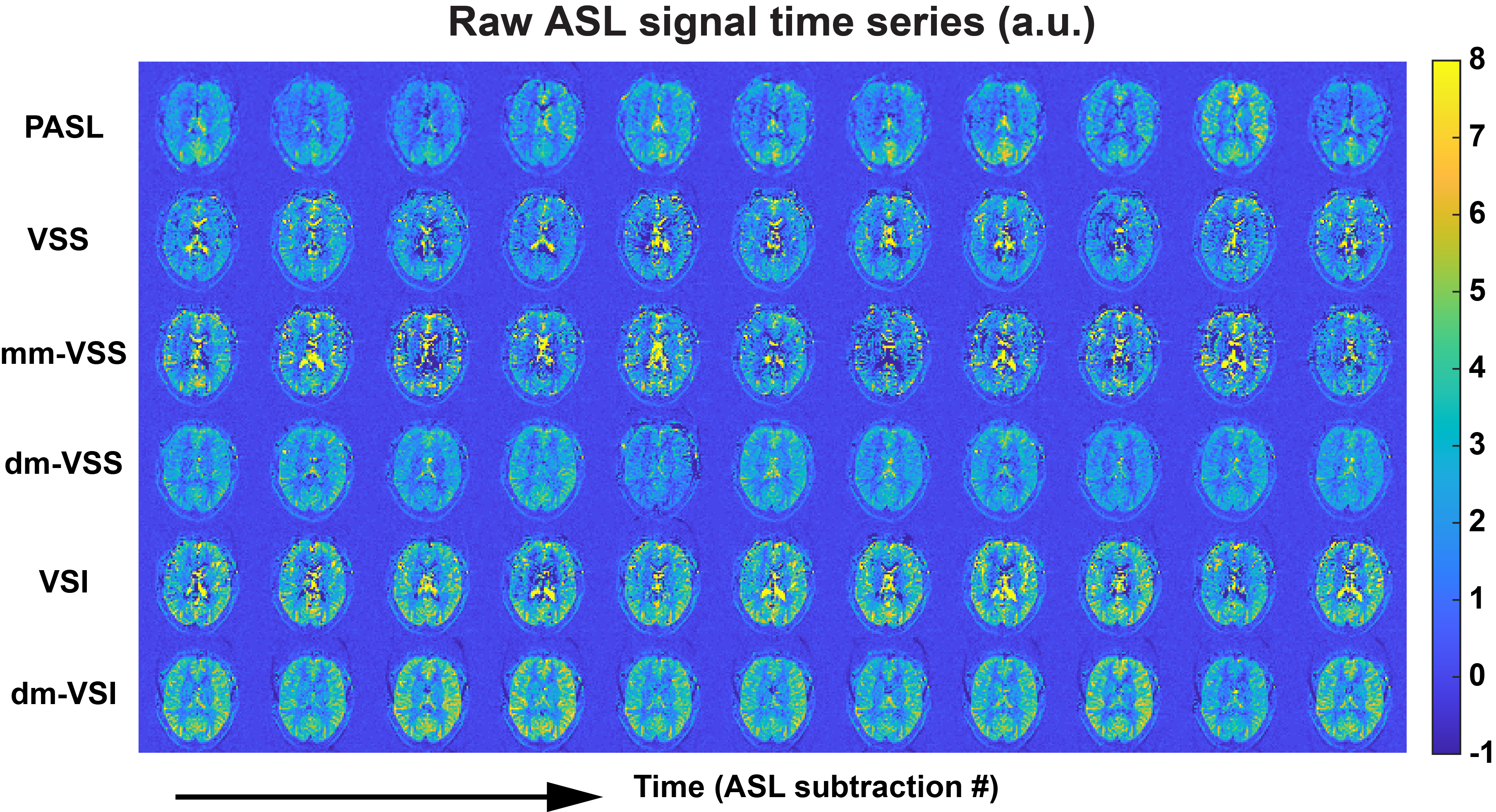
**

**Supporting Information Figure S2**: Representative raw ASL signal time series from Subject 2 (only the first 11 time points are shown for PASL), the acquisition time for each image was 16 s for PASL and 20 s for VSASL. Note the superior stability of labeling using dm-VSS and dm-VSI across time.

**Derivation of dm-VSASL signal to include T_2_ relaxation and β**

To have a more precise picture of the VSASL signal generation, we have to properly introduce the T_2_ relaxation and β at the right time, and with the assumption that β affects the magnetization under both the label and control conditions. Now we can track the magnetization evolution during the preparation process.

1. Taking **dm-VSI** as an example:

**For the group of arterial spins that is labeled only by the first VSI module (Group 1):**

The arterial longitudinal magnetization of the label image right after the first VSI module:

$M_{z1,lab}=M_{0}\cdot(1-e^{-\frac{T_{sat}}{T_{1a}}})\cdot\beta_{VSI}\cdot e^{-\frac{{eTE}_{VSI}}{T_{2a}}}$,

And that of the control image:

$M_{z1,con}=-M_{0}\cdot(1-e^{-\frac{T_{sat}}{T_{1a}}})\cdot\beta_{VSI}\cdot e^{-\frac{{eTE}_{VSI}}{T_{2a}}}$;

After including the T_1_ relaxation during TI_1_, and the application of the second VSI module (note that the module inverts the magnetization in both the label and control images, and only T_2_ relaxation is in effect since this group has arrived at capillaries):

$M_{z1,lab}=-M_{0}\cdot e^{-\frac{{eTE}_{VSI}}{T_{2a}}}\cdot(1-(1-(1-e^{-\frac{T_{sat}}{T_{1a}}})\cdot\beta_{VSI}\cdot e^{-\frac{{eTE}_{VSI}}{T_{2a}}})\cdot e^{-\frac{{TI}_{1}}{T_{1a}}})$,

And for the control image:

$M_{z1,con}=-M_{0}\cdot e^{-\frac{{eTE}_{VSI}}{T_{2a}}}\cdot(1-(1+(1-e^{-\frac{T_{sat}}{T_{1a}}})\cdot\beta_{VSI}\cdot e^{-\frac{{eTE}_{VSI}}{T_{2a}}})\cdot e^{-\frac{{TI}_{1}}{T_{1a}}})$;

Then after including the T_1_ relaxation during TI_2_,

$\Delta M_{z1}=M_{z1,con}-M_{z1,lab}=2M_{0}\cdot e^{-\frac{{eTE}_{VSI}}{T_{2a}}}\cdot\alpha_{VSI}\cdot e^{-\frac{{TI}_{1}+{TI}_{2}}{T_{1a}}}\cdot(1-e^{-\frac{T_{sat}}{T_{1a}}})$, where $\alpha_{VSI}=\beta_{VSI}\cdot e^{-\frac{{eTE}_{VSI}}{T_{2a}}}$.

**For the group of arterial spins that is labeled by both VSI modules (Group 2):**

After the first VSI module:

$M_{z2,lab}=M_{0}\cdot(1-e^{-\frac{T_{sat}}{T_{1a}}})\cdot\beta_{VSI}\cdot e^{-\frac{{eTE}_{VSI}}{T_{2a}}}$,

And that of the control image:

$M_{z2,con}=-M_{0}\cdot(1-e^{-\frac{T_{sat}}{T_{1a}}})\cdot\beta_{VSI}\cdot e^{-\frac{{eTE}_{VSI}}{T_{2a}}}$;

After the application of the second VSI module (note the label/control switching, and that both T_2_ relaxation and β are in effect):

$M_{z2,lab}=-M_{0}\cdot\beta_{VSI}\cdot e^{-\frac{{eTE}_{VSI}}{T_{2a}}}\cdot(1-(1-(1-e^{-\frac{T_{sat}}{T_{1a}}})\cdot\beta_{VSI}\cdot e^{-\frac{{eTE}_{VSI}}{T_{2a}}})\cdot e^{-\frac{{TI}_{1}}{T_{1a}}})$,

And for the control image:

$M_{z2,con}=M_{0}\cdot\beta_{VSI}\cdot e^{-\frac{{eTE}_{VSI}}{T_{2a}}}\cdot(1-(1+(1-e^{-\frac{T_{sat}}{T_{1a}}})\cdot\beta_{VSI}\cdot e^{-\frac{{eTE}_{VSI}}{T_{2a}}})\cdot e^{-\frac{{TI}_{1}}{T_{1a}}})$;

Then after including the T_1_ relaxation during TI_2_,

$\Delta M_{z2}=M_{z2,con}-M_{z2,lab}=2M_{0}\cdot\alpha_{VSI}\cdot e^{-\frac{{TI}_{2}}{T_{1a}}}\cdot(1-e^{-\frac{{TI}_{1}}{T_{1a}}})$.

2. For **dm-VSS using VSS_inv_+VSS**, the magnetization of spins of **Group 1** after the first VSS_inv_ module:

$M_{z1,lab}=0$,

And $M_{z1,con}=-M_{0}\cdot(1-e^{-\frac{T_{sat}}{T_{1a}}})\cdot\beta_{VSS}\cdot e^{-\frac{{eTE}_{VSS}}{T_{2a}}}$;

And the magnetization after the second VSS module:

$M_{z1,lab}=M_{0}\cdot e^{-\frac{{eTE}_{VSS}}{T_{2a}}}\cdot(1-e^{-\frac{{TI}_{1}}{T_{1a}}})$,

And $M_{z1,con}=M_{0}\cdot e^{-\frac{{eTE}_{VSS}}{T_{2a}}}\cdot(1-(1+(1-e^{-\frac{T_{sat}}{T_{1a}}})\cdot\beta_{VSS}\cdot e^{-\frac{{eTE}_{VSS}}{T_{2a}}})\cdot e^{-\frac{{TI}_{1}}{T_{1a}}})$;

So $\Delta M_{z1}=M_{z1,con}-M_{z1,lab}=-M_{0}\cdot e^{-\frac{{eTE}_{VSS}}{T_{2a}}}\cdot\alpha_{VSS}\cdot e^{-\frac{{TI}_{1}+{TI}_{2}}{T_{1a}}}\cdot(1-e^{-\frac{T_{sat}}{T_{1a}}})$, where $\alpha_{VSI}=\beta_{VSS}\cdot e^{-\frac{{eTE}_{VSS}}{T_{2a}}}$.

For arterial spins of **Group 2**:

The magnetization of spins of Group 2 after the first VSS_inv_ module:

$M_{z2,lab}=0$,

And $M_{z2,con}=-M_{0}\cdot(1-e^{-\frac{T_{sat}}{T_{1a}}})\cdot\beta_{VSS}\cdot e^{-\frac{{eTE}_{VSS}}{T_{2a}}}$;

After the second VSS module (note the label/control switching):

$M_{z2,lab}=M_{0}\cdot\beta_{VSS}\cdot e^{-\frac{{eTE}_{VSS}}{T_{2a}}}\cdot(1-e^{-\frac{{TI}_{1}}{T_{1a}}})$,

and $M_{z2,con}=0$;

so $\Delta M_{z2}={M'}_{z2,con}-{M^{'}}_{z2,lab}=-M_{0}\cdot\alpha_{VSS}\cdot e^{-\frac{{TI}_{2}}{T_{1a}}}\cdot(1-e^{-\frac{{TI}_{1}}{T_{1a}}})$.

3. Similarly, for **hybrid dm-VSASL using VSI+VSS**,

$\Delta M_{z1}=-2M_{0}\cdot e^{-\frac{{eTE}_{VSS}}{T_{2a}}}\cdot\alpha_{VSI}\cdot e^{-\frac{{TI}_{1}+{TI}_{2}}{T_{1a}}}\cdot(1-e^{-\frac{T_{sat}}{T_{1a}}})$,

and $\Delta M_{z2}=-M_{0}\cdot\alpha_{VSS}\cdot e^{-\frac{{TI}_{2}}{T_{1a}}}\cdot(1-e^{-\frac{{TI}_{1}}{T_{1a}}}+\alpha_{VSI}\cdot e^{-\frac{{TI}_{1}}{T_{1a}}}-\alpha_{VSI}\cdot e^{-\frac{T_{sat}+{TI}_{1}}{T_{1a}}})$.

4. And for **mm-VSS**:

$\Delta M_{z1}=M_{0}\cdot e^{-\frac{{eTE}_{VSS}}{T_{2a}}}\cdot\alpha_{VSS}\cdot e^{-\frac{{TI}_{1}+{TI}_{2}}{T_{1a}}}\cdot(1-e^{-\frac{T_{sat}}{T_{1a}}})$,

and $\Delta M_{z2}=M_{0}\cdot\alpha_{VSS}\cdot e^{-\frac{{TI}_{2}}{T_{1a}}}\cdot(1-e^{-\frac{{TI}_{1}}{T_{1a}}}+\alpha_{VSS}\cdot e^{-\frac{{TI}_{1}}{T_{1a}}}-\alpha_{VSS}\cdot e^{-\frac{T_{sat}+{TI}_{1}}{T_{1a}}})$.
